# Standard dose and low-dose rituximab combined with glucocorticoids and cyclophosphamide in the treatment of acquired haemophilia A

**DOI:** 10.3389/fimmu.2025.1642982

**Published:** 2025-08-29

**Authors:** Mingxia Cai, Xizhe Guo, Xueya Zhang

**Affiliations:** ^1^ Department of Internal Medicine, Second Affiliated Hospital of Fujian Medical University, Quanzhou, Fujian, China; ^2^ Department of Hematology, Second Affiliated Hospital of Fujian Medical University, Quanzhou, Fujian, China

**Keywords:** acquired haemophilia A, rituximab, low dose, standard dose, immunosuppressive therapy

## Abstract

**Aim:**

To investigate the efficacy and safety of standard dose and low-dose rituximab combined with corticosteroids and cyclophosphamide in the treatment of acquired haemophilia A.

**Methods:**

A retrospective analysis was conducted on the clinical manifestations, laboratory tests, diagnosis and treatment process, and efficacy of 11 patients with acquired haemophilia A.

**Results:**

Among the 11 patients, there were 6 males and 5 females with median age 64 years old (29-88 years); 11 patients had no underlying diseases before onset. Clinical manifestations include varying degrees of skin bruising, with 1 case accompanied by muscle hematoma, 1 case accompanied by gastrointestinal bleeding, and 1 case accompanied by abdominal hematoma; The laboratory examination showed an APTT prolongation of 85.97 ± 27.26 seconds, which could not be corrected after incubation with normal plasma for 2 hours; FVIII: C activity 0.93 ± 1.51%; The titer of FVIII inhibitor is 15.19 ± 14.32BU; All 11 patients received a combination of prothrombin complex and fresh frozen plasma for hemostasis, with 1 patient receiving a single platelet transfusion; Among the 11 patients, 7 received a standard dose of rituximab (375mg/m2/w × 1w) combined with corticosteroids and cyclophosphamide regimen, and 4 received a low-dose rituximab (100mg/w × 4w) combined with corticosteroids and cyclophosphamide regimen. The APTT recovery time was 37.3 ± 8.6 days, 43.6 ± 11.4 days (P>0.05), and the FVIII activity increased to normal time was 30.2 ± 9.8 days, 40.2 ± 18.8 days (P<0.05), respectively; The time for FVIII inhibitor to turn negative was 56.3 ± 23.5 days and 63.9 ± 29.1 days, respectively (P<0.05); Four weeks after treatment, there was no statistically significant difference in the levels of B lymphocyte antigen, IgG, IgA, IgM, and incidence of adverse reactions between the two groups (P>0.05).

**Conclusions:**

Acquired haemophilia A is rare in clinical practice, with severe and common bleeding manifestations. Fresh frozen plasma combined with prothrombin complex had a good hemostatic effect. The standard dose of rituximab combined with corticosteroids and cyclophosphamide can quickly increase FVIII activity and eradicate FVIII inhibitors compared to the low dose of rituximab regimen, although there is no difference in efficacy and safefy between the two treatment regimens.

## 1 Introduction

Acquired haemophilia A (AHA) refers to a rare bleeding disorder caused by the production of specific inhibitors of endogenous coagulation factor VIII in patients with normal hemostatic function, leading to a decrease in plasma FVIII activity (1–3). It mostly occurs in patients with malignant tumors, autoimmune diseases, and perinatal women, but half patients are idiopathic (4–6).

The treatment principles for AHA are to control bleeding as early as possible and to clear inhibitors as soon as possible (7–9). Rituximab has been increasingly used for the treatment of inhibitor clearance in patients with AHA. However, the optimal medication regimen for rituximab is still uncertain at present (10, 11). We retrospectively analyzed 11 patients with AHA who were treated with conventional doses of rituximab combined with cyclophosphamide and corticosteroids, as well as low-dose rituximab combined with cyclophosphamide and corticosteroids. We analyzed the clearance time of inhibitors, recovery of coagulation factor VIII activity levels, humoral immune recovery time, and incidence of infection in these patients.

## 2 Methods

### 2.1 Patients

Retrospective analysis was conducted on 11 patients with AHA admitted between January 2018 and December 2024. Informed consent was obtained from the patients and approved by the Fujian Medical University, Second Hospital ethics committee.

### 2.2 Diagnostic criteria

Patients with AHA meet the following criteria: ① There is a tendency for bleeding, but no history of bleeding or family history of bleeding; ② The activated partial thromboplastin time is prolonged, and the incubation of l: l plasma at 37 °C for 2 hours cannot completely correct it, while the prothrombin time and thrombin time are normal; ③ The FVIII level was measured using one-stage assay, the Bethesda method was used to determine the titer of FVIII inhibitors or the effectiveness of immunosuppressive therapy.

### 2.3 Differential diagnosis

All patients undergo routine examinations such as blood routine, liver and kidney function, immune screening (complement c3, complement C4, anti nuclear antibodies, anti double stranded DNA antibodies, anti SM, anti SSA, anti SSB and anti cardiolipin antibodies, lupus anticoagulant, etc.), tumor markers, abdominal ultrasound, chest X-ray or chest and abdominal CT to understand the underlying disease of the patient.

### 2.4 Treatment plan

The treatment for patients with AHA includes hemostatic therapy and inhibitor clearance therapy.

1. Hemostasis treatment: according to the patient’s bleeding symptoms, given that the high cost of recombinant factor VIIa (rFVIIa) and emicizumab, as well as the unavailability of activated prothrombin complex (aPCC), fresh frozen plasma and prothrombin complex have been infused.
2. Inhibition clearance therapy: including glucocorticoids, cyclophosphamide, and rituximab.

### 2.5 Efficacy evaluation

Complete remission: bleeding symptoms have been controlled, FVIII: C rises to normal, and no inhibitors can be detected (<0.6BU);

Partial remission: bleeding symptoms have been improved, F VIII: C increased by more than 50%, and inhibitor droplets decreased by 50%;

Invalid treatment: bleeding symptoms have not been improved, F VIII: C remains low, and antibody levels are high;

The treatment of bleeding are determined by the size of the hematoma, the stability of hemoglobin/hematocrit, and the degree of pain caused by the hematoma.

### 2.6 Statistical analysis

SPSS 28.0 software was used to perform statistical analysis on the obtained data. The patient count data was analyzed using a chi square test, and the measurement data followed a normal distribution, expressed as mean ± standard deviation. The inter group comparison was analyzed using a t-test, while the non normal distribution data was analyzed using a median. The inter group comparison was analyzed using a non parametric test. P<0.05 indicates that the difference is statistically significant.

## 3 Results

### 3.1 Patient characteristics

Among them, there were 6 males and 5 females with median age 64 years old (29-88 years). There were no significant differences in disease onset time, gender, age, clinical manifestations, underlying diseases, etc. between patients receiving conventional doses of rituximab combined with cyclophosphamide and corticosteroids and those receiving low-dose rituximab combined with cyclophosphamide and corticosteroids (**Table 1**).

**Table 1:** Basic characteristics between two groups of patients.

Basic characteristics		Standard dose rituximab combined with cyclophosphamide and glucocorticoids (7 cases)	Low dose rituximab combined with cyclophosphamide and glucocorticoids (4 cases)	χ2	P
Gender	male	4	2	0.16	0.68
	female	3	2		
Age(years)		53 ± 20	67 ± 20	1.54	0.21
Date of onset (in days)		9.3 ± 3.5	11.3 ± 4.3	0.38	0.54
Multiple skin bleeding		7	4	0.19	0.66
Abdominal hematoma		1	0	0.14	0.71
Gastrointestinal bleeding		1	0	0.14	0.71
Underlying disease		1	1	0.18	0.67
PT(s)		13.5 ± 1.6	13.6 ± 1.6	0.60	0.43
APTT(s)		85.9 ± 27.3	71.6 ± 16.8	0.34	0.56
vWF(%)		168.3 ± 40.7	157.9 ± 27.7	0.62	0.43
WBC(×10^9^/L)		9.4 ± 2.9	9.5 ± 1.3	0.54	0.46
HGB(g/L)		86.3 ± 11.9	83.5 ± 13.4	0.67	0.41
PLT(×10^9^/L)		265.6 ± 66.5	305.5 ± 80.1	1.27	0.26
VIII Activity(%)		0.93 ± 1.51	0.45 ± 0.41	0.73	0.39
VIII Inhibitor Titer(BU)		15.2 ± 14.3	9.2 ± 8.3	0.09	0.76

### 3.2 Treatment measures

#### 3.2.1 Hemostasis

All 11 patients were treated with infusion of fresh frozen plasma and prothrombin complex; One patient with abdominal hematoma was given fresh platelet transfusion (**Table 2**).

**Table 2:** Hemostasis and inhibitor clearance protocol between two groups of patients.

Patient	Hemostasis plan	Clearing inhibitor protocol
1	Fresh frozen plasma, prothrombin complex	Methylprednisolone (1mg/kg/d) combined with cyclophosphamide (1mg/kg/d) and rituximab 375mg/m2 intravenously, once a week for a total of once
2	Fresh frozen plasma, prothrombin complex	Methylprednisolone (1mg/kg/d) combined with cyclophosphamide (1mg/kg/d) and rituximab 375mg/m2 intravenously, once a week for a total of once
3	Fresh frozen plasma, prothrombin complex + platelet	Methylprednisolone (1mg/kg/d) combined with cyclophosphamide (1mg/kg/d) and rituximab 375mg/m2 intravenously, once a week for a total of once
4	Fresh frozen plasma, prothrombin complex	Methylprednisolone (1mg/kg/d) combined with cyclophosphamide (1mg/kg/d) and rituximab 375mg/m2 intravenously, once a week for a total of once
5	Fresh frozen plasma, prothrombin complex	Methylprednisolone (1mg/kg/d) combined with cyclophosphamide (1mg/kg/d) and rituximab 375mg/m2 intravenously, once a week for a total of once
6	Fresh frozen plasma, prothrombin complex	Methylprednisolone (1mg/kg/d) combined with cyclophosphamide (1mg/kg/d) and rituximab 375mg/m2 intravenously, once a week for a total of once
7	Fresh frozen plasma, prothrombin complex	Methylprednisolone (1mg/kg/d) combined with cyclophosphamide (1mg/kg/d) and rituximab 375mg/m2 intravenously, once a week for a total of once
8	Fresh frozen plasma, prothrombin complex	Methylprednisolone (1mg/kg/d) combined with cyclophosphamide (1mg/kg/d) and rituximab 100mg/d, once a week, for a total of 4 times
9	Fresh frozen plasma, prothrombin complex	Methylprednisolone (1mg/kg/d) combined with cyclophosphamide (1mg/kg/d) and rituximab 100mg/d, once a week, for a total of 4 times
10	Fresh frozen plasma, prothrombin complex	Methylprednisolone (1mg/kg/d) combined with cyclophosphamide (1mg/kg/d) and rituximab 100mg/d, once a week, for a total of 4 times
11	Fresh frozen plasma, prothrombin complex	Methylprednisolone (1mg/kg/d) combined with cyclophosphamide (1mg/kg/d) and rituximab 100mg/d, once a week, for a total of 4 times

#### 3.2.2 Clearing coagulation factor inhibitors

11 cases of methylprednisolone (1-2mg/kg/d) combined with cyclophosphamide (2mg/kg/d); Among them, 4 patients were treated with rituximab 100mg/d once a week for a total of 4 times; Seven patients received intravenous infusion of 375mg/m2 of rituximab in combination, once a week for a total of one time (**Table 2**).

### 3.3 Efficacy

After treatment, all 11 patients showed improvement in clinical symptoms, with 9 cases (81.8%) achieving complete remission and 2 cases (18.2%) experiencing partial remission; The complete response rates of patients receiving conventional doses of rituximab combined with cyclophosphamide and glucocorticoids and low-dose rituximab combined with cyclophosphamide and glucocorticoids were 6 cases (85.7%) and 3 cases (75%), respectively; The clinical bleeding relief time was 21.7 ± 6.6 days and 25.7 ± 7.3 days, respectively; The recovery time of APTT to normal was 37.3 ± 8.6 days and 43.6 ± 11.4 days, respectively; The time for a 50% decrease in F VIII inhibitor titer was 28.5 ± 8.7 days and 33.7 ± 9.6 days, respectively. The time for a 50% increase in F VIII activity was 23.8 ± 7.3 days and 35.2 ± 10.2 days, and the time for F VIII activity to return to normal was 30.2 ± 9.8 days and 40.2 ± 18.8 days, respectively; The conversion time of FVIII inhibitor to negative was 56.3 ± 23.5 days and 63.9 ± 29.1 days, respectively (**Table 3**).

**Table 3:** The therapeutic effect between two groups of patients.

Therapeutic efficacy	Standard dose rituximab combined with cyclophosphamide and corticosteroids (7 cases)	Low dose rituximab combined with cyclophosphamide and glucocorticoids (4 cases)	χ2	P
Clinical bleeding relief time	7.7 ± 1.6	9.7 ± 2.3	1.13	0.28
APTT recovery time to normal	37.3 ± 8.6	43.6 ± 11.4	1.19	0.27
Time for FVIII activity to increase by 50%	23.8 ± 7.3	35.2 ± 10.2	4.81	0.03
FVIII activity recovery time to normal	30.2 ± 9.8	40.2 ± 18.8	4.91	0.03
FVIII inhibitor reduced by 50%	28.5 ± 8.7	33.7 ± 9.6	2.72	0.09
FVIII inhibitor conversion time	56.3 ± 23.5	63.9 ± 29.1	5.32	0.02
Efficacy assessment	6 cases were completely remission, and 1 case was partially remission	3 cases were completely remission, and 1 case was partially remission	–	0.64

### 3.4 B lymphocyte antigen expression and immunoglobulin levels

There were used to evaluate the impact on humoral immunity between patients receiving conventional doses of rituximab combined with cyclophosphamide and corticosteroids and those receiving low-dose rituximab combined with cyclophosphamide and corticosteroids; We analyzed the antigen expression and immunoglobulin levels of B lymphocytes before and 4 weeks after medication. The results showed that there was no significant difference in the levels of B lymphocyte antigen, IgG, IgM, and IgA between the two groups (P>0.05) (**Table 4**).

**Table 4:** B lymphocyte antigen expression and immunoglobulin levels between two groups of patients.

B cell antigen expression and immunoglobulin levels	Standard dose rituximab combined with cyclophosphamide and corticosteroids (7 cases)		Low dose rituximab combined with cyclophosphamide and glucocorticoids (4 cases)		*P*
	Before treatment	After treatment	Before treatment	After treatment	
B cell antigen (%)	18% ± 4%	14% ± 3%	11.2% ± 2.2%	11% ± 1.6%	0.73
IgG (g/L)	13.4 ± 1.5	9.5 ± 1.2	12.8 ± 0.9	11.5 ± 1.1	0.3
IgM (g/L)	1.3 ± 0.4	1.1 ± 0.3	1.5 ± 0.2	1.3 ± 0.2	0.38
IgA (g/L)	2.5 ± 0.9	1.7 ± 0.8	2.9 ± 0.2	2.6 ± 0.3	0.06

### 3.5 Adverse reactions

Among the 11 patients with adverse reactions, those who received conventional doses of rituximab combined with cyclophosphamide and glucocorticoids experienced fever, nausea, vomiting, pneumonia, and hyperglycemia. Patients receiving low-dose rituximab combined with cyclophosphamide and glucocorticoids therapy also have fever, pneumonia, and hyperglycemia (**Table 5**).

**Table 5:** Adverse reactions between two groups of patients.

Adverse reactions	Standard dose rituximab combined with cyclophosphamide and corticosteroids (7 cases)	Low dose rituximab combined with cyclophosphamide and glucocorticoids (4 cases)	*P*
Fever	2	1	1
Nausea	1	0	1
Vomit	1	0	1
Pneumonia	1	1	1
Rash	0	0	1
Shortness of breath	0	0	1
Joint pain	0	0	1
High blood sugar	1	1	1

### 3.6 Survival analysis

As of December 31, 2024, all 11 patients were still alive. All patient’s APTT and FVllI levels would be reexamined once a month within 6 months, every 2-3 months from the 6th to the 12th month, and every 6 months after the 2nd year. Among them, 3 patients relapsed, including one patient after received low-dose rituximab combined with cyclophosphamide and glucocorticoids therapy 12 months and two patients after received conventional doses of rituximab combined with cyclophosphamide and glucocorticoids therapy 18 months, respectively, showed by an increase in antibody titers and a decrease in coagulation factor VIII activity levels and accompanied by bleeding. After treatment with rituximab combined with cyclophosphamide and glucocorticoids, complete remission were achieved again.

## 4 Discussion and conclusions

Acquired haemophilia A (AHA) is a rare disease caused by depletion or inhibition of coagulation factors due to autoantibodies (12–15). Its clinical characteristics, treatment, and prognosis are significantly different from those of hereditary haemophilia (16, 17). Among coagulation factor inhibitors, factor VIII (FVIII) inhibitors that cause AHA have been widely reported. In AHA, FVIII inhibitory antibodies neutralize FVIII activity and cause bleeding. If not treated properly, it may lead to life-threatening bleeding complications with a mortality rate as high as 9% -41% (18–20).

Most AHA patients have no clear cause. The onset of AHA in some patients may be related to autoimmune diseases, pregnancy and childbirth, malignant tumors, drug allergies, skin diseases, and respiratory system diseases, but the mechanism by which these underlying diseases lead to AHA is still unclear (21). Among the 11 patients in this group, 2 patients had relatively clear etiology and were both related to connective tissue disease. According to literature reports, for AHA patients with concomitant autoimmune diseases, the most common autoimmune disease is systemic lupus erythematosus (SLE), followed by rheumatoid arthritis (RA). In this group of patients, bleeding was the initial presentation, and 2 cases were diagnosed with AHA combined with connective tissue disease. This suggests that the production of coagulation factor VIII inhibitors may be related to the activity level of underlying diseases such as connective tissue disease (22).

The treatment of AHA includes primary disease treatment, hemostatic treatment, and clearance of coagulation factor inhibitors (23). Treating the primary disease is a prerequisite for completely controlling AHA, and hemostatic treatment is the key to AHA treatment. For patients with obvious bleeding tendency, both domestic and foreign guidelines recommend recombinant FVIIa and activated prothrombin complex as first-line medications. Unlike hereditary haemophilia, due to the presence of inhibitors of coagulation factor VIII, the hemostatic effect of coagulation factor VIII preparations is not precise. Currently, there are no prospective randomized controlled trials at home and abroad to confirm the effectiveness of supplementing recombinant human coagulation factor VIII preparations. The efficacy of recombinant FVIIa in AHA patients is influenced by multiple factors such as disease type, bleeding site, and severity. The application of recombinant FVIIa in the early stages of bleeding in AHA patients is crucial for therapeutic efficacy. At the same time as hemostatic treatment, underlying diseases should be actively treated. However, due to the high cost of recombinant FVlIa and emicizumab, as well as the unavailability of activated prothrombin complex (aPCC), fresh frozen plasma and prothrombin complex were used for hemostasis treatment in all cases of this group. Patient 3 in this group developed severe abdominal hematoma accompanied by severe anemia. Although platelet count was normal, thromboelastography showed a significant decrease in MA value, reflecting abnormal platelet function in the patient. Considering the risk of bleeding, fresh platelet transfusion was given. From the perspective of clinical efficacy, hemostatic treatment is ineffective before the start of coagulation factor inhibitor clearance therapy. There are literature reports that some patients with low titers of coagulation factor inhibitors can achieve relief by receiving hemostatic treatment. For those with high titers, only receiving hemostatic treatment cannot achieve relief. Clearing coagulation factor inhibitors is still the key to AHA treatment.

To eradicate FVIII inhibitory antibodies, effective first-line IST is to use steroids alone or in combination with cyclophosphamide. There is no prospective comparison between the use of steroids alone and the combination therapy of steroids and cyclophosphamide; A retrospective study conducted in the UK showed no difference between the two groups, while the European Acquired Haemophilia Registry (EACH2) study and meta-analysis showed that the steroids and cyclophosphamide combination treatment group had faster remission. According to previous reports, the remission rate of using steroids alone is 30% -48%, which is lower than the 60% -90% of steroids and cyclophosphamide combination therapy; But when cyclophosphamide is used, the frequency of adverse reactions such as infection increases, leading to an increase in mortality rate. If the above treatment fails, rituximab can be used, with a response rate of 59% -90% (16, 17).

Rituximab is a monoclonal antibody drug with therapeutic potential for clearing coagulation factor inhibitors (24, 25). There have been reports both domestically and internationally of the use of rituximab (20, 21, 25), with a recommended dose of 375 mg/(m^2^/week), administered once a week for a total of 4 times, achieving complete remission in the treatment of refractory AHA. According to the recently published article, meta-analysis of the data published from 1981 to 2024 indicated Cyclophosphamide plus steroid regimen and rituximab-only regimen had similar efficacy in terms of complete remission and lower relapse rate. They concluded adding rituximab to the standard regimen did not produce superior outcomes. It is suggested to add a third group with standard regimen (cyclophosphamide plus steroids) without Rituximab and compare three groups in safety and efficacy (26). However, there is a lack of large-scale research data to confirm its efficacy and dosage, and further confirmation is needed.

Hunt S et al. reported (27) that a low dose rituximab regimen (100mg weekly for 4 weeks) was used in 6 patients with AHA as efficacious as standard dose rituximab regimen (375mg/m2 weekly for 4 weeks) for 4 AHA patients. While the other study by Fu YH et al. (28) demonstrated that 6 AHA cases achieved complete remission by lowered dose of rituximab (rituximab: 100 mg weekly × 4 or 500 mg once in week 1) and bortezomib in combination with corticosteroids and cyclophosphamide regimen, suggesting this regimen is feasible treatment for AHA. However, we retrospectively analyzed the effectiveness of clearing inhibitors in 11 patients. Seven patients received a standard dose of rituximab (375mg/m2 weekly for 1 week) combined with cyclophosphamide and corticosteroids regimen, while four patients received a low-dose rituximab (100mg weekly for 4 weeks) combined with cyclophosphamide and corticosteroids regimen. The results showed that compared with the low-dose regimen group, the standard dose regimen group increased FVIII activity faster, shortened FVIII inhibitor conversion time, and had shorter hemostasis time. Moreover, in terms of adverse reactions, we analyzed the antigen expression and immunoglobulin levels of B lymphocytes before and 4 weeks after medication. There was no significant difference in the levels of B lymphocytes and immunoglobulin IgG, IgM and IgA between the two groups. These results have not been described in previous studies on the same topic (27, 28). And then, fever and pneumonia were common adverse events, but after symptomatic treatment and supplementation with human immunoglobulin, they were all controlled, indicating that the standard dose and low dose regimen groups had good safety.

In summary, AHA is a dangerous condition, and improving bleeding symptoms and clearing inhibitors are the key to successful treatment of AHA patients. Our retrospective analysis indicated that the standard dose rituximab or low dose rituximab combined with cyclophosphamide and corticosteroids regimen are effective treatment for AHA, and with no difference in side effects. Moreover, the standard dose regimen group increased FVIII activity faster, shortened FVIII inhibitor conversion time. Considering the limitations of retrospective studies, the selection of treatment regimens may be affected by patients’ own conditions, leading to “treatment bias”, thus, it cannot verify the real effect of intervention measures like randomized cotrolled trials. And other, the insufficient sample size of AHA cases may easily result in insufficient stabiligy of the results, it is necessary to include more cases for prospective research to confirm.

## Data Availability

The original contributions presented in the study are included in the article/Supplementary Material. Further inquiries can be directed to the corresponding author.
